# Uncoupling between cerebral perfusion and oxygenation during incremental exercise in an athlete with postconcussion syndrome: a case report

**DOI:** 10.14814/phy2.13131

**Published:** 2017-01-25

**Authors:** Sarah Imhoff, Simon Malenfant, Éric Nadreau, Paul Poirier, Damian M. Bailey, Patrice Brassard

**Affiliations:** ^1^ Department of KinesiologyFaculty of MedicineLaval UniversityQuebec CityQuebecCanada; ^2^ Research Center of the Institut universitaire de cardiologie et de pneumologie de QuébecLaval UniversityQuebec CityQuebecCanada; ^3^ Pulmonary Hypertension Research GroupQuebec Heart and Lungs Institute Research CenterLaval UniversityQuebec CityQuebecCanada; ^4^ Neurovascular Research LaboratoryFaculty of Life Sciences and EducationUniversity of South WalesSouth WalesUnited Kingdom; ^5^ Sondes Moléculaires en BiologieLaboratoire Chimie Provence UMR 6264 CNRSUniversité de Provence MarseilleMarseilleFrance

**Keywords:** Cerebral oxygenation, cerebral perfusion, exercise, postconcussion syndrome

## Abstract

High‐intensity exercise may pose a risk to patients with postconcussion syndrome (PCS) when symptomatic during exertion. The case of a paralympic athlete with PCS who experienced a succession of convulsion‐awakening periods and reported a marked increase in postconcussion symptoms after undergoing a graded symptom‐limited aerobic exercise protocol is presented. Potential mechanisms of cerebrovascular function failure are then discussed.

## Introduction

Mild traumatic brain injury (mTBI) or concussion is at the beginning of the continuum of damage severity following a TBI. Although recovery following mTBI is usually a short‐lived process resolving after 7–10 days, it has recently been postulated that alterations in cerebrovascular function, especially cerebral autoregulation (CA) and cerebrovascular reactivity to carbon dioxide (CVR), could partly contribute to the presence of postconcussion symptoms (Len and Neary 2011; Amonette and Mossberg 2013; Bailey et al. 2013; Len et al. 2013; Conder and Conder 2014; Gardner et al. 2014b; Tan et al. 2014; Clausen et al. 2015; Kenney et al. 2015; Meier et al. 2015; Sours et al. 2015; Svaldi et al. 2015). Deficits in CA and CVR have been identified in acute and chronic mTBI as well as following moderate‐to‐severe TBI in humans (Dewitt and Prough 2003; Len and Neary 2011; Gardner et al. 2014b; Tan et al. 2014; Kenney et al. 2015). The use of low‐to‐moderate intensity aerobic exercise has been proposed as a promising therapeutic approach when postconcussion symptoms persist beyond the expected recovery period (Leddy et al. 2007, 2012; Archer et al. 2012; Baker et al. 2012; Mccrory et al. 2013; Schneider et al. 2013; Silverberg and Iverson 2013; Zemek et al. 2014). Moreover, symptom‐limited incremental exercise emerged as a safe tool to assess recovery and guide return‐to‐play decision‐making in postconcussed athletes (Leddy et al. 2010, 2011; Leddy and Willer 2013; Tan et al. 2014). Conversely, aerobic exercise may trigger the appearance or exacerbation of postconcussion symptoms, which could decrease exercise tolerance in patients with postconcussion syndrome (PCS) (Leddy et al. 2010; Kozlowski et al. 2013). Autoregulatory deficits may contribute to exercise‐induced symptoms through excessive changes in cerebral blood flow (CBF) (Archer et al. 2012; Clausen et al. 2015) and could make the brain more vulnerable to hypo‐ and/or hyperperfusion during and after high‐intensity exercise.

Whether high‐intensity exercise poses a risk to patients with PCS when symptomatic during exertion remains to be determined. We report herein the case of a paralympic athlete with PCS who experienced a succession of convulsion‐awakening periods and reported a marked increase in postconcussion symptoms following an incremental exercise protocol. We then briefly review the potential mechanisms of cerebrovascular function failure that may explain this case.

## Case Report

A 35‐year‐old woman competing in Tricycle Class T2 (e.g. UCI Para‐cycling Classification Guide) with hemiplegia of the left limb induced by transversal myelitis treated with fludrocortisone, pregabalin and codeine consulted our services because of a history of exercise‐induced headache, dizziness and syncope subsequent to a concussion caused by a bicycle fall in 2012. While still symptomatic from this first concussion suffered in 2012, the patient experienced worsening of symptoms consecutive to a subsequent fall caused by a loss of consciousness at the end of a strenuous bout of exercise in a competition in August 2014. At the time of the consultation, she reported headaches, neck pain, dizziness, tonic‐clonic movements post‐high intensity exercise, balance problems, difficulties concentrating and memory problems for more than 1 month following mTBI, which is consistent with the World Health Organisation (ICD‐10) definition of PCS. Rest and gradual return to activity was therefore recommended as proposed by major guidelines of sport‐related concussion management, and was treated by an interdisciplinary team specialized in the management of patients with mTBI (Giza et al. 2013; Harmon et al. 2013; Mccrory et al. 2013; Ontario Neurotrauma Fondation, 2013, Marshall et al. 2015). Five months later, after being asymptomatic at rest and returned to normal activities and function (work/submaximal exercise training), she attempted an incremental exercise protocol at the *Institut Universitaire de Cardiologie et de Pneumologie de Québec* (IUCPQ), aiming at identifying the cause of effort‐related syncope.

The patient was requested to be well rested, be fasting, well hydrated, abstain from caffeinated beverages or stimulants and alcohol for 2 h prior to the assessment as well as refrained from strenuous exercise for 24 h. The patient performed a stepwise incremental upright cycling protocol carried out on a LeMond Revolution training device equipped with the WattBox system. Intensity was gradually increased every 2 min for the first 4 levels (from 30 to 56 W) and then every minute (from 65 to 220 W) up to exhaustion. Breath‐by‐breath pulmonary gas exchange was monitored using an automated gaz analyzer (Ultima™, CardiO2^®^ gas exchange analysis system; MGC Diagnostics^®^, MN, USA) with the athlete breathing through a mouthpiece attached to a pneumotachometer, for determination of oxygen consumption (V̇O_2_), carbon dioxide production (V̇CO_2_), respiratory exchange ratio (RER: V̇CO_2_/V̇O_2_), ventilation and end‐tidal partial pressure of carbon dioxide (P_ET_CO_2_). Maximal exercise values were defined as the highest 60‐s averaged values. Middle cerebral artery mean blood flow velocity (MCAvmean) was monitored by transcranial Doppler ultrasound (TCD) (Doppler Box; Compumedics DWL USA, Inc. San Juan Capistrano, CA), through the left temporal window as described by Willie et al. (2011a). Near‐infrared spectroscopy (NIRS) was used to measure left frontal cerebral oxygenation (ScO_2_) (Oxiplex TS; ISS, Champlain, IL, USA). ScO_2_ was calculated by the ratio of oxygenated hemoglobin (HbO_2_) on total hemoglobin [HbO_2_ + deoxygenated hemoglobin (HHb)]: HbO_2_/(HbO_2_ + HHb) × 100. Heart rate (HR) was continuously monitored by a 12‐lead electrocardiogram (ECG) and blood pressure taken manually every 2 min using a sphygmomanometer. Steady‐state baseline data were monitored at rest in test position for 3 min. Data (MCAvmean and ScO_2_) were sampled at 1 kHz using an analog‐to‐digital converter (Powerlab 16/35; ADInstruments), and time‐aligned with breath‐by‐breath measurements, for offline analyses. Data were then averaged over 60 sec up to maximal exercise.

**Figure 1:**
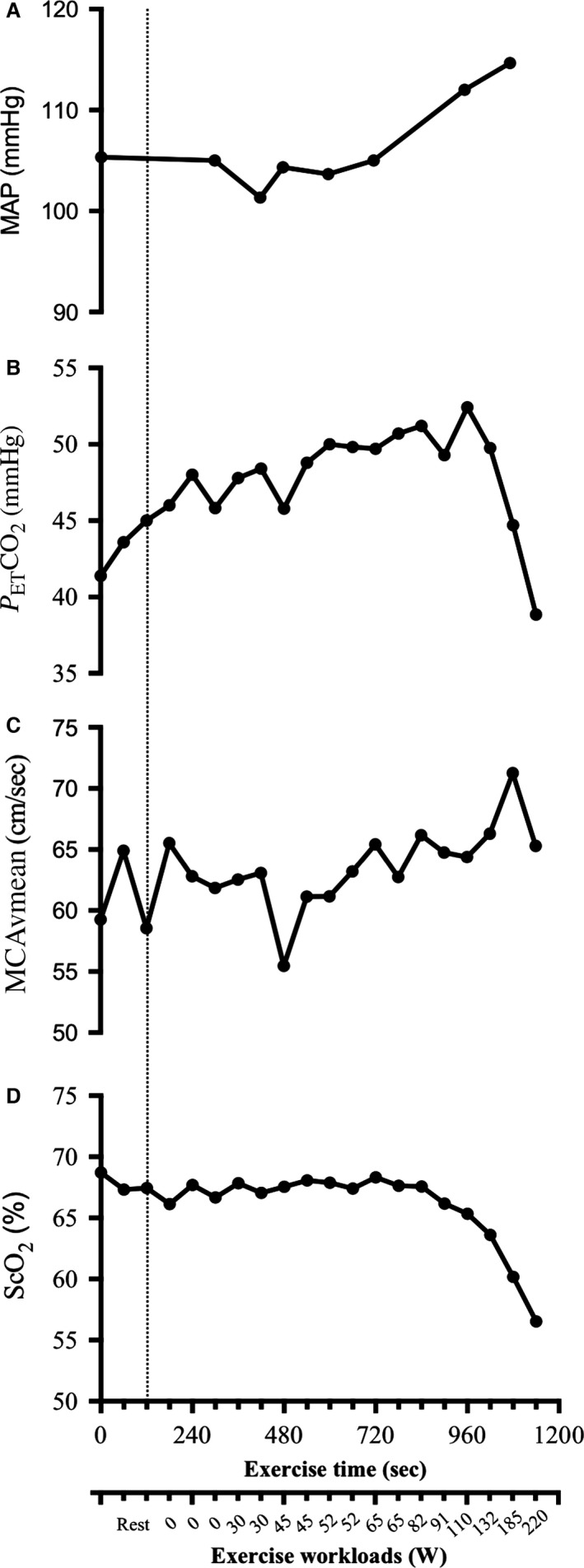
Mean arterial pressure (MAP) (A), End‐tidal partial pressure of carbon dioxide (P_ET_CO_2_) (B), middle cerebral artery mean blood flow velocity (MCAvmean) (C), and frontal cerebral oxygenation (ScO_2_) (D) in function of time (sec) during exertion test and exercise workloads (W). Vertical dotted line represents the beginning of exercise.

MCAvmean increased by 20% and did not return to baseline values notwithstanding the presence of hyperventilation‐induced hypocapnia at higher exercise intensities (Fig. 1). Despite this increase in cerebral perfusion, ScO_2_ lowered by 18% during the last 5 min of the exercise protocol (>10% decrease in the last 2 min).

The athlete reported headache during the last stages of the exercise protocol. Cardiorespiratory variables reached at maximal voluntary exercise are presented in Table 1. At test cessation, the patient lost consciousness and experienced a succession of convulsion‐awakening periods with tonic‐clonic movements. She was therefore transferred to the emergency room of the IUCPQ, where she was monitored on electroencephalogram, which remained normal. Thereafter, she was transferred to a specialized affiliate hospital in the field of neurology. A thorough investigation included electroencephalography, two lumbar punctures, as well as computed tomography cerebral angiography and cerebral magnetic resonance studies. At the end of the investigation, the final diagnosis was nonepileptic seizure.

**Table 1:** Cardiorespiratory variables at maximal voluntary exercise

	Maximal values
Heart rate (bpm)	186
Ventilation (L·min^−1^)	105
Peak oxygen consumption (mL·kg^−1^·min^−1^)	49.07
Peak oxygen consumption (L·min^−1^)	2.64
Respiratory exchange ratio	1.33

Respiratory exchange ratio: carbon dioxide production/oxygen consumption.

## Discussion

Cerebral blood flow regulation is complex and its main determinants are arterial pressure, arterial blood gases, neurovascular coupling and the autonomic nervous system (Ogoh and Ainslie 2009; Willie et al. 2014). To our knowledge, cerebrovascular responses to exercise in individuals recovering from mTBI have only been explored briefly specifically in individuals with PCS. This patient had a history of exertion‐induced headache, dizziness and syncope, which is consistent with symptoms reported in athletes with PCS experiencing exercise intolerance (Leddy et al. 2010; Kozlowski et al. 2013; Clausen et al. 2015). Cerebral autoregulation failure has been proposed as a possible contributor to the appearance or exacerbation of exercise‐induced postconcussion symptoms through critical changes in CBF (lowered CBF following arterial hypotension or increased CBF following arterial hypertension) (Leddy et al. 2010; Archer et al. 2012; Kozlowski et al. 2013; Gardner et al. 2014a; Toth et al. 2016). For instance, recent findings from an animal study have demonstrated that TBI is associated with an important vasodilation of cerebral arteries induced by an elevation in nitric oxide production, which in turn leads to a loss in myogenic tone, an important determinant of cerebral autoregulation (Villalba et al. 2014). Moreover, impairments in CVR have been reported in repetitive impact sports (Bailey et al. 2013; Svaldi et al. 2015, 2016), PCS (Mutch et al. 2014, 2015; Chan et al. 2015; Clausen et al. 2015) and acute mTBI (Len et al. 2011, 2013; Militana et al. 2015). Impairments in important determinants of CBF regulation could not only contribute to the presence of postconcussion symptoms such as cognitive dysfunction, headache and dizziness during exercise (Len and Neary 2011; Amonette and Mossberg 2013; Bailey et al. 2013; Len et al. 2013; Conder and Conder 2014; Gardner et al. 2014b; Tan et al. 2014; Clausen et al. 2015; Kenney et al. 2015; Meier et al. 2015; Sours et al. 2015; Svaldi et al. 2015) but could make the brain more vulnerable to hypoperfusion and hyperperfusion during and after high‐intensity exercise which may pose a risk to patients with PCS when symptomatic during exertion.

**Figure 2:**
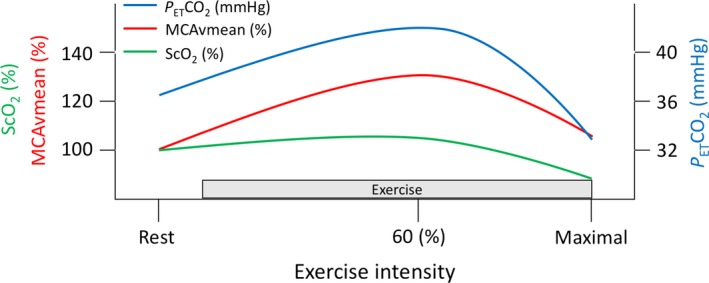
Schematic representation of the normal responses in middle cerebral artery mean blood flow velocity (MCAvmean; red trace), cerebral oxygenation (ScO_2_; green trace), and end‐tidal partial pressure of carbon dioxide (P_ET_CO_2_; blue trace) during incremental exercise to exhaustion.

We noticed an unusual uncoupling between changes in P_ET_CO_2_ and MCAvmean during the incremental exercise protocol, e.g. an increase in cerebral perfusion notwithstanding hyperventilation‐induced hypocapnia (see Fig. 2 for normal responses in MCAvmean, P_ET_CO_2_ and ScO_2_ to incremental exercise to volitional exhaustion). This finding could be explained by an attenuated CVR, which has already been described in PCS (Chan et al. 2015; Clausen et al. 2015). Despite this increase in cerebral perfusion, ScO_2_ decreased by 18%, although ScO_2_ usually increases slightly in athletes during submaximal exercise (Brugniaux et al. 2014). We speculate that the incapacity of exercise‐induced hypocapnia to dampen changes in MCAvmean, coupled to a less efficient CA, may eventually lead to an autoregulatory breakthrough and a breakdown of the blood‐brain barrier resulting in extracellular vasogenic edema. For example, patients with hyperperfusion syndrome may present with headache, seizures, focal neurological deficits, visual abnormalities and nausea/vomiting (Neki and Mani 2013; Fugate and Rabinstein 2015), which is consistent with symptoms induced by strenuous exercise experienced by this patient. The presence of regional cerebral edema may then exacerbate tissue O_2_ diffusion limitation leading to a mismatch between delivery and higher cerebral metabolism during heavy exercise, which in turn would lower cerebral oxygenation (Ogoh and Ainslie 2009). Of note, increased metabolic demand subsequent to mTBI (Giza et al. 2013) could increase the risk of mismatch between oxygen supply and demand in the brain during aerobic exercise. Importantly, a 10–15% reduction in ScO_2_ has been associated with presyncope in healthy individuals (Colier et al. 1997; Madsen et al. 1998; Harms et al. 2000; Kay and Rickards 2015). However, since changes in CBF seem to be greater in the brainstem compared to the cortex during alterations in PaCO_2_ and PaO_2_ (Willie et al. 2012), exercise (Willie et al. 2011b) and during central hypovolemia (Kay and Rickards 2015), we cannot rule out the possibility that a reduction in blood flow in the posterior cerebral circulation may be responsible for the reduction in ScO_2_ and exercise‐induced syncope in this patient.

Therefore, exercise‐induced symptoms may reflect cerebrovascular function impairments and should be taken into consideration when assessing functional responses to exercise in individuals with PCS. High‐intensity exercise should be introduced carefully in this population as recommended by major guidelines.

## Conclusion

Although exercise may be beneficial when recovering from mTBI, it should be introduced carefully. Post‐concussion cerebrovascular function impairments could make the brain more vulnerable to hypo/hyperperfusion during and after high‐intensity exercise.

## Conflict of Interest

The authors have no conflict of interest.
